# Perspective: Clinical care of pedophilic individuals in Zurich, Switzerland

**DOI:** 10.1038/s41443-024-00968-6

**Published:** 2024-08-29

**Authors:** Fanny de Tribolet-Hardy, Simon Veitz, Laura Dittli, Elmar Habermeyer

**Affiliations:** University of Zurich, Department of Forensic Psychiatry, University Hospital of Psychiatry Zurich, Zurich, Switzerland

**Keywords:** Health care, Risk factors

## Abstract

Individuals with pedophilia are considered to have an elevated risk for child sexual abuse (CSA). Nevertheless, it is assumed that pedophilic sexual impulses can be controlled from acting out. To prevent CSA an outpatient treatment facility for people with pedophilia was founded in Zurich, Switzerland in 2021. The program focuses on the prevention of CSA and improvement of quality of life, incorporating empirically validated treatment principles, such as the Risk-Need-Responsivity (RNR) model and the Good Lives Model (GLM). Within the initial 24-month 142 individuals sought help, 46 individuals (mean age 36.0 ± 12.4 years) completed the assessment phase, two-thirds suffered from psychiatric comorbidities, and 67.4% reported sexual delinquency. The high drop-out rate was predominantly due to ongoing criminal proceedings, or other mental health conditions. Overall patients at this facility generally sought treatment voluntarily, leading to good treatment adherence, with severe self-harm being more prevalent than acute danger to others. A treatment approach focused solely on pedophilia is considered insufficient; psychiatric and psychosocial factors must also be addressed.

## Introduction

Child sexual abuse (CSA) is one of the most serious and far-reaching offenses. Meta-analyses considering self-reports by victims suggest that sexual abuse affects approximately 13.4% to 19.7% of girls and 5.7% to 8.8% of boys worldwide [1, 2]. These high victimization rates are not reflected in police crime statistics, thus a large dark field is assumed [3]. Empirical studies on child sexual abusers show that only a proportion (25–50%) have a pedophilic disorder [4, 5]. This subgroup is nevertheless prognostically relevant: Offenders with an exclusive pedophilic disorder have a higher risk of recidivism than those without pedophilic disorder [6, 7].

Pedophilia refers to sexual interest in pre- or early pubescent persons, whereas hebephilia is a sexual interest in pubescent persons. The prevalence of pedophilia is estimated at around 1% in general population, with men being affected more frequently than women [4].

Individuals with pedophilia therefore represent a group at risk for offending. Although studies of persons with pedophilic interests outside the justice system are scarce, it is assumed that, analogous to other sexual preferences, pedophilic sexual impulses can be controlled from acting out or even the interest itself might be modified [8–10]. In treatment, «control» of the pedophilic interest focuses on awareness and regulation of potentially destabilizing / risk-increasing factors (e.g., substance use, stress, cognitive distortions). Besides, recent empirical studies suggest the existence of a specific self-efficacy in pedophilic individuals reflecting their beliefs about their ability to modify their pedophilic interest. This concept might be relevant for motivational processes and the possibility of actual changes [9]. Nevertheless, surveys show that a substantial proportion of individuals with sexual interests in children reported not having committed illegal acts [11]. In this respect, pedophilia and child sexual abuse should not be used synonymously.

In addition to crime prevention, however, there is another aspect to consider: Studies point to a high need for therapeutic support among this clientele with a significantly increased suicidal tendency—especially in the case of additional psychological comorbidities, own experiences of abuse, perceived stigmatization due to the paraphilic tendency, and increased exclusivity of the paraphilic interest [11, 12]. However, empirical studies show that qualified therapists are rare, which is attributed to pronounced resentment towards this clientele and a lack of professional expertise [13]. In this perspective, Niehaus et al. argue that prevention of subjective suffering and the improvement of well-being and quality of life should also be a secondary prevention goal [13].

The present perspective outlines the development and implementation of a treatment program for pedophilic individuals in Switzerland. We then focus on describing the current patients participating in this program, their treatment needs, and the difficulties of reaching these patients.

## Development in Switzerland

Establishing treatment services for individuals with pedophilic disorder is not new in Europe. Its origins can be found in Hamburg, Germany from the 1960s on [14]. From 2005 on, the high-profile project “Kein Täter Werden” (meaning *do not offend*) by the Institute for Sexual Science and Sexual Medicine of the Charité Berlin improved accessibility to the target group [15]. Because of the increasing consumption of Child Sexual Abuse Material (CSAM) further programs have been developed that focus on the accessibility of CSAM-offenders, whereby online treatment services, including on the darknet, have been established [16].

In Switzerland, secondary prevention measures gained political importance in 2016 when, in addition to a revision of the law governing sexual offenses, the evaluation of prevention strategies was advocated. Based on the findings by Niehaus et al. [13], the Swiss government (Federal Council) recommended in 2020 the establishment of treatment centers and specific training for professionals. Subsequently, with the support of the Department of Health in the Canton of Zurich, the Pedosexuality Preventive Services was established at the Psychiatric University Hospital Zurich in May 2021. Funding was provided as a three-year pilot project, taking free-of-cost access with anonymous contact and counseling into account. Both measures are considered relevant in order to reach this highly stigmatized group of patients [13]. In June 2021, the association “Kein Täter Werden Suisse” was founded with other treatment locations in the cantons of Basel, Thurgau, and Geneva. The Pedosexuality Prevention Services in Zurich remains nevertheless the only treatment location in Switzerland to receive financial support.

## Implementation

The Pedosexuality Preventive Services are affiliated with the Clinic for Forensic Psychiatry due to its primarily focus on crime prevention. It was set up in collaboration with the Institute for Sex Research, Sexual Medicine and Forensic Psychiatry of the University Clinic Hamburg-Eppendorf, Germany and by involving different cantonal directorates (Department of Health, Department of Justice, Department of Education). This enabled to counter any concerns at an early stage and to promote broad political acceptance. Cross-directorate meetings were maintained during the project and facilitated to establish important synergies with police and child protection. Issues related to the treatment of anonymous persons proved to be relevant. In particular, concerns about how to deal with possible endangerment of children lead to the development of corresponding guidelines with a step-by-step model for reporting endangerment of others to the criminal prosecution. For the purpose of reaching the target group the website www.kein-taeter-werden.ch was created, which serves as an information and contact portal. Other strategies include presentations, advertising campaigns on public transport, and social media.

## Assessment and treatment

Psychoeducational content and primary therapy goals are conveyed during the initial consultation. The central idea of the “Kein Täter Werden” network is that although there is no one to blame for their pedophilic preference, they must take responsibility for the resulting behavior [8]. The diagnostic phase includes a detailed assessment of biographical and sexual history, psychological tests, and a risk assessment [14]. In this phase, comorbidities are assessed and any necessary somatic investigations are carried out, such as urological examinations or blood tests. The aim is to develop a holistic case concept. The subsequent treatment follows a strongly individualistic approach whereby comorbidities, as well as empirically validated treatment principles such as the risk-need-responsivity principle [17] and the Good Lives Model [18] are taken into account. In addition, pharmaceutical treatment options are carried out based on the guidelines for pharmacological treatment of paraphilic disorders [19].

## Data

The presented data in Table 1 was collected as part of a quality management process during the diagnostic phase. We used semi-structured interviews and questionnaires, such as the BDI-II [20], SCID-5-PD [21], and HBI [22], to assess sexual preference patterns, sexual behavior, and psychiatric comorbidities. The diagnoses were assigned according to the criteria of the ICD-10 or DSM-5 - except for hypersexuality the criteria by Kafka was used (A: Min. 6 months of recurrent and intense sexual fantasies, urges, behaviors fulfilling ≥3 of the following criteria: 1. Interference with other important (non-sexual) obligations; 2. As a response to dysphoric mood states; 3. As a response to stressful life events; 4. Unsuccessful attempts at reduction; 5. Disregard of the risk for physical or emotional harm to self or others. B: Clinically significant personal distress or impairment. C: No direct physiological effect of an exogenous substance). If available, we considered mental health reports provided by our patients. The forensic data was obtained during an interview and is based on self-report.

**Table 1:** Clinical information (*N* = 46^a^).

Sexual preference^b^	*n* (%)
Non-exclusive pedophilic/hebephilic	40 (87.0%)
…thereof pedophilic	10 (21.7%)
… thereof hebephilic	17 (37.0%)
… thereof pedo-and hebephilic	13 (28.3%)
Exclusive pedophilic/hebephilic	6 (13.0%)
… thereof pedophilic	5 (10.9%)
… thereof hebephilic	1 (2.2%)
Sexual behavior^c^	*n* (%)
Masturbation to pedophilic and/or hebephilic fantasies	41 (89.1%)
Use of legal images of children	25 (54.3%)
Use of CSAM^d^	20 (43.5%)
Online contact with children	14 (30.4%)
CSA^e^	0 (0.0%)
Other psychiatric disorders^f^	*n* (%)
No other psychiatric disorder	17 (37.0%)
One psychiatric comorbidity	20 (43.5%)
Two or more psychiatric comorbidities	9 (19.6%)
Affective disorders	13 (28.3%)
Personality disorders	11 (23.9%)
Substance use disorders	4 (8.7%)
Sexual dysfunction disorders	3 (6.5%)
Other paraphilic disorders	2 (4.4%)
Anxiety disorders	2 (4.4%)
Attention deficit hyperactivity disorder	2 (4.4%)
Hypersexuality	2 (4.4%)
Asperger syndrome	1 (2.2%)
Developmental disorders	1 (2.2%)
Forensic information^g^	*n* (%)
No report of CSA or Use of CSAM	15 (32.6%)
CSA or/and Use of CSAM unknown to the penal system	19 (41.3%)
At least one CSA or/and Use of CSAM conviction	12 (26.1%)

^a^Total sample;

^b^Self-report by patients, diagnostic criteria according to DSM-5;

^c^Self-report by patients in first assessment; multiple responses possible;

^d^*CSAM* Child Sexual Abuse Material;

^e^*CSA* Child Sexual Abuse;

^f^According to the criteria of the ICD-10 or DSM-5 except for hypersexuality, which was diagnosed using the criteria by Kafka (23);

^g^Self-report by patient.

**Fig. 1:**
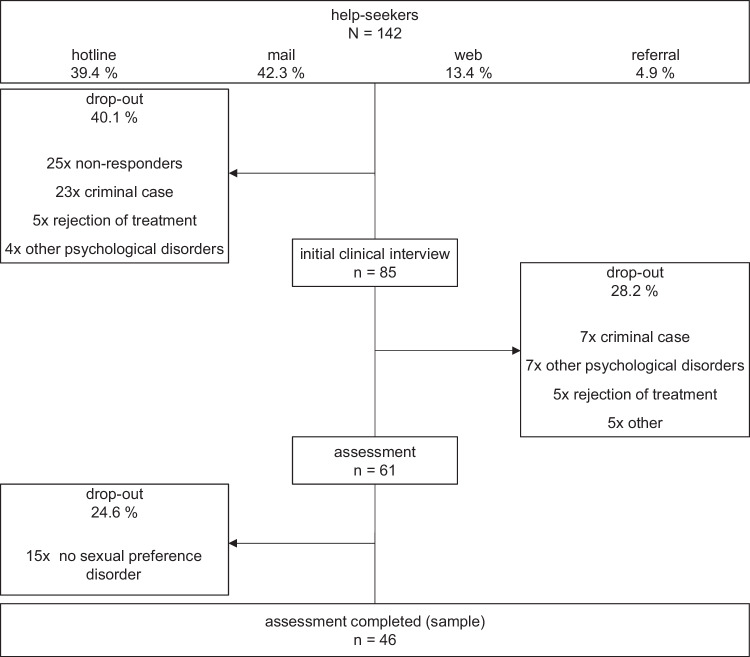
Recruitment Process. Illustration of the recruitment process: 46 of the 142 people seeking help remained after initial clinical interview and assessment.

The data relates to a 24-month period from the start of the project in June 2021 till June 2023. Of 142 persons seeking help (136 m / 4 f / 2 unknown), 46 (45 m / 1 f) persons completed the assessment phase. The overall drop-out rate was 67.6% (Fig. 1). The mean age of the final sample (*n* = 46) was 36.0 ± 12.4 years (mean age of men was 36.4 ± 12.5 whereas the female patient was 22.0 years old).

Concerning sexual preference patterns a majority described a non-exclusive preference pattern with pedophilic and/or hebephilic preferences (Table 1). The minority reported an exclusive pedophilic or hebephilic disorder. Almost two-thirds of the sample suffered from one or more psychiatric comorbidities. In terms of forensic data, 67.4% reported sexual delinquency. 63.9% of the reported delinquent acts were unknown to the penal system (47.2% CSAM, 16.7% CSA), whereas 36.1% had been convicted (22.2% CSAM, 13.9% CSA). The data relies fully on self-report.

## Discussion

Research emphasizes that persons with pedophilic interests are among the most stigmatized group of individuals in society [23]. This is important when reaching out to this patient group, as the psychological stress associated with stigmatization (fear of discovery of pedophilic interests, perceived loneliness, emotion regulation problems, low self-esteem) causes reduced help-seeking and is assumed to be an indirect risk factor for the commission of sexual abuse [24, 25]. In line with this, the pilot project has shown that providing low-threshold and anonymized treatment is essential for reaching this highly stigmatized patient group. Especially the fact that no information is passed on to health insurance companies was considered essential by our patients. In order to guarantee this, financial funding is necessary. Despite many initial concerns about anonymous treatment and associated potential risks related to offending, all of our patients disclosed their personal information to the practitioners during assessment, which illustrates a high level of trust in the practitioners and in the handling of personal data.

Furthermore, a comprehensive concept, as advocated by the “Kein Täter Werden” network, is considered relevant in order to reach this group of patients but also to create public acceptance for a (funded) treatment offer. In this context, the differentiation between the sexual preference (the fate of the person affected) and the associated problematic or delinquent behavior (the responsibility of the person affected) is highly relevant.

Our data indicates high drop-out rates. Reasons for dropping out (40.1%) after first contact were criminal proceedings, other severe mental illnesses, or refusal of an appointment. In 25 cases, the circumstances remained unknown: In 24 of the 25 cases, contact was made by e-mail without further correspondence. One patient was referred by a specialist without further contact. Concerning the drop-out rate after the initial consultation, our data is comparable to similar treatment locations [26]. Our data suggests that the majority of the drop-outs can be attributed to formal exclusion criteria (ongoing criminal proceedings / other psychiatric disorders).

Most of the patients at our facility seek treatment voluntarily, which is why good treatment adherence is common. Aspects of severe self-harm were more frequent than aspects of acute danger to others. A report to the police due to potential danger to others has not been filed yet. In line with reportings from similar programs by other institutes [27], this highlights that criminal prevention in terms of hands-on-delinquency is the most important, but not the only goal in treatment for this group of patients. Most of our patients told us, that they never committed CSA (s. Table 1); rather, the consumption of CSAM, comorbidities, and clinically relevant psychological distress are their predominant treatment needs. This illustrates that a treatment program aimed exclusively at pedophilia is insufficient [28]. Psychiatric comorbidities and psychosocial stress factors as potential indirect risk factors for committing sexual abuse must also be taken into account and be evaluated during treatment. Potential outcome variables of treatment need to be assessed on various dimensions, such as the reduction of specific behavior (e.g., frequency of CSAM use), but also potential indirect risk factors, such as e.g., psychosocial functioning and treatment outcomes concerning other psychiatric comorbidities. This individualistic approach results in a high degree of heterogeneity in treatment but is in line with common forensic treatment concepts and crime prevention [17, 18, 29].

Finally, further research should also address critical points: There is no reliable empirical data on whether this approach actually has a crime prevention effect in terms of preventing CSA or CSAM [30]. Empirical studies have considerable methodological shortcomings (no control group, small sample size, self-report, etc.). Nevertheless, web-based treatments show empirical evidence for a reduction in CSA and use of CSAM [31].

Secondly, concerns about possible negative (risk-increasing) effects of forensic sex offender treatment should be addressed. Such effects have been shown to be particularly relevant for offenders with a low risk of reoffending [32]. From our point of view, the treatment received in our program cannot be compared with criminal justice measures. Our patients seek treatment voluntarily and are mostly intrinsically motivated. They have more opportunities to influence the course of treatment (frequency, length of treatment, content) and probably show a greater openness in reporting problematic behavior (especially use of CSAM, online contact with children) to their therapist due to not having to fear negative consequences (e.g., reporting to authorities). Nevertheless, such dynamics need to be evaluated during treatment by taking the mentioned aspects into account, as e.g., openness concerning problematic behavior, motivational factors, and the impact of psychiatric comorbidities on the risk of offending.

## Data Availability

The presented data was collected within a quality management process.
